# Crystal Structure of a 9-Subunit Archaeal Exosome in Pre-Catalytic States of the Phosphorolytic Reaction

**DOI:** 10.1155/2012/721869

**Published:** 2012-12-20

**Authors:** Esben Lorentzen, Elena Conti

**Affiliations:** Department of Structural Cell Biology, Max Planck Institute of Biochemistry, Am Klopferspitz 18, 82152 Martinsried, Germany

## Abstract

The RNA exosome is an important protein complex that functions in the 3′ processing and degradation of RNA in archaeal and eukaryotic organisms. The archaeal exosome is functionally similar to bacterial polynucleotide phosphorylase (PNPase) and RNase PH enzymes as it uses inorganic phosphate (Pi) to processively cleave RNA substrates releasing nucleoside diphosphates. To shed light on the mechanism of catalysis, we have determined the crystal structures of mutant archaeal exosome in complex with either Pi or with both RNA and Pi at resolutions of 1.8 Å and 2.5 Å, respectively. These structures represent views of precatalytic states of the enzyme and allow the accurate determination of the substrate binding geometries. In the structure with both Pi and RNA bound, the Pi closely approaches the phosphate of the 3′-end nucleotide of the RNA and is in a perfect position to perform a nucleophilic attack. The presence of negative charge resulting from the close contacts between the phosphates appears to be neutralized by conserved positively charged residues in the active site of the archaeal exosome. The high degree of structural conservation between the archaeal exosome and the PNPase including the requirement for divalent metal ions for catalysis is discussed.

## 1. Introduction

RNA exosomes are key players in degradation, processing, and quality control of a wide variety of RNA molecules [1] and have a structurally conserved 9-subunit core common to eukarya and archaea [2]. The common exosome core is composed of a hexameric ring of RNase PH subunits (Rrp41 and Rrp42 in archaea) capped on one side by three protein subunits containing RNA binding domains (Rrp4 and Csl4 in archaea) [3]. This architecture results in a barrel-like complex with a continuous central channel implicated in RNA binding in both archaea [4] and eukarya [5–7]. Although the core architecture of exosome complexes is conserved, the mechanisms of RNA degradation have diverged substantially [8, 9]. Whereas the archaeal exosome has an active phosphorolytic RNase PH core [10, 11], eukaryotic exosomes rely on the additional hydrolytic RNAses Rrp44 [12–14] and Rrp6 [15–17] for activity. Interestingly, the phosphorolytic activity of the archaeal exosome is reversible resulting in the decay of RNAs in the presence of Pi as well as in the addition of polynucleotide tails in the presence of nucleotide diphosphates, an activity that has also been shown to occur *in vivo* [18, 19]. The activity of the archaeal exosome is, thus, more similar to that of the bacterial polynucleotide phosphorylase (PNPase) [20, 21] than to eukaryotic exosomes, a notion further supported by the fact that residues involved in substrate binding and catalysis are well conserved among archaeal exosome and PNPase complexes [10, 22].

A number of crystal structures have been determined of archaeal exosomes in both apo and RNA bound forms from *Sulfolobus solfataricus* [4, 10, 22, 23], *Pyrococcus abyssi* [24], *Archaeoglobus fulgidus* [3], and *Methanothermobacter thermautotrophicus* [25]. These structures have revealed the overall architecture of the complexes and visualized RNA at the active site as well as inside the central channel. Additionally, the structure of the *M. thermautotrophicus* exosome core revealed the presence of one inorganic phosphate (Pi) ion bound at the active site of the complex [25]. The general framework for RNA binding at the phosphorolytic site of the archaeal exosome is, thus, well understood. However, little is known about the reaction mechanism, mainly because of the absence of structures of reaction intermediates. To this end, we determined the crystal structure of a 9-subunit *S. solfataricus* exosome mutant in complex with both RNA and Pi. This structure represents a precatalytic complex prior to the nucleophilic attack by the phosphate leading to 3′-end cleavage of the RNA substrate. Based on this structure, we present a model for the archaeal exosome highlighting the importance of divalent cations in catalysis.

## 2. Experimental Procedures

### 2.1. Crystal Soaking, X-Ray Diffraction Data Collection, and Structure Solution

The *S. solfataricus* exosome was recombinantly expressed in *E. coli*, purified and crystallized as previously described [4, 27]. The 1.8 Å resolution structure with one Pi ion bound was obtained after soaking crystals with 1 mM 7 mer poly(A) RNA and 10 mM Pi for 20 min followed by flash cooling in liquid nitrogen. Electron density maps calculated at 1.8 Å resolution revealed very clear density for the Pi ion but only spurious density for RNA. The 2.5 Å resolution structure with RNA and Pi bound was obtained by first soaking crystals for 48 h with 1 mM 7 mer poly(A) RNA followed by a 20 min soak with 10 mM Pi and immediate flash cooling in liquid nitrogen. X-ray diffraction data were collected at the Swiss Light Source beamline X06SA and processed with the program XDS [28]. The structure was determined using the available Rrp4/41/42 structure as a starting model followed by iterative cycles of refinement in REFMAC (Pi bound structure) [29] or PHENIX (RNA∗Pi bound structure) [30] and model building in COOT [31]. All figures were made in the program PyMOL (http://www.pymol.org/).  Figure 4(a) was prepared by superposing the native Rrp41/42 structure (pdb code 2BR2) onto Rrp4/41^D182A^/42 mutant exosome to obtain the conformation of the D182 side chain. The position of the divalent cation was obtained by superimposing the coordinating aspartates (D486 and D492) of *E. coli* PNPase structure (pdb code 3GME) onto the equivalent residues (D182 and D188) of the *S. solfataricus* exosome.

## 3. Results and Discussion

### 3.1. High-Resolution Structure of the Phosphate Binding Site in Rrp41

**Figure 1:**
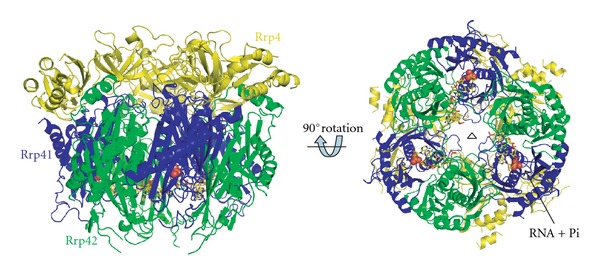
Overall structure of the *S. solfataricus* exosome with the two RNase PH subunits Rrp41 and Rrp42 displayed in blue and green, respectively, and the RNA binding protein Rrp4 displayed in yellow. The bound RNA substrate is shown as a stick model and the inorganic phosphate atoms as spheres. The picture on the right shows a 90-degree rotation around the horizontal axis with the 3-fold symmetry axis indicated as a triangle.

To trap a complex of the archaeal exosome with Pi and RNA substrates bound at the active site, nonameric 3∗(Rrp41/Rrp42/Rrp4) complex from *S. solfataricus* with the D182A point mutation in the Rrp41 protein (Rrp41^D182A^) was used. This point mutation was previously shown to completely abolish RNase activity but is not directly involved in RNA or phosphate binding and presumably allows for both substrates to bind without being turned over [10, 22]. Rrp4/Rrp41^D182A^/Rrp42 mutant exosome crystallized in the cubic space group P213 with one copy of each subunit in the asymmetric unit and the full 9-subunit complex is generated by a crystallographic 3-fold axis (Figure 1). Pi and RNA were soaked into crystals that were cooled in liquid nitrogen at various time points resulting in the determination of a crystal structure of the archaeal exosome at 1.8 Å resolution with Pi bound at the active site and a 2.5 Å resolution structure where both RNA and Pi are bound at the active site (see Table 1 for data collection and refinement statistics).

**Figure 2:**
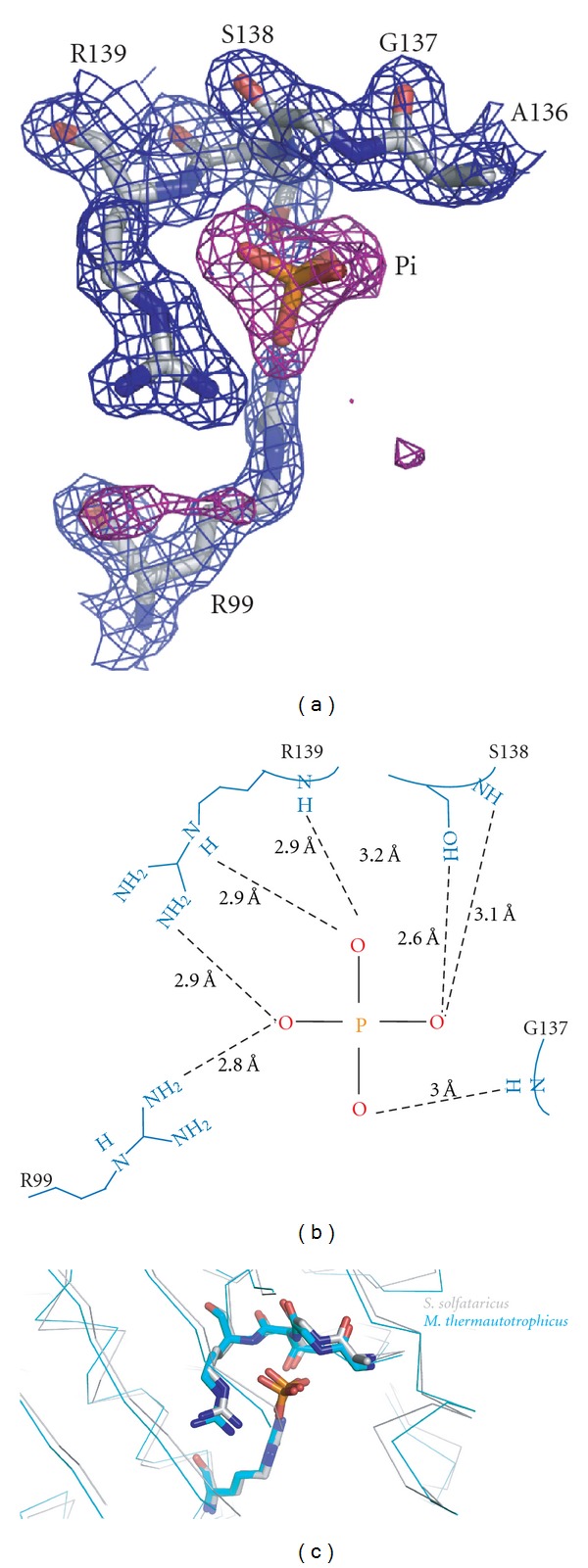
High-resolution view of the inorganic phosphate binding site. (a) Unbiased Fo-Fc map at 3 sigma is shown in magenta and 2Fo-Fc map at 1 sigma in blue. The inorganic phosphate and contacting residues from the Rrp41 subunit are shown as sticks. (b) Schematic representation of the inorganic phosphate binding site with distances between the Pi ion and protein residues indicated. (c) Structures of the *S. solfataricus* and the *M. thermautotrophicus* exosomes superimposed reveal that the Pi-binding sites are very well conserved across different archaeal species.

Previously determined crystal structures of *S. solfataricus *exosomes crystallized in the absence of Pi displayed spherical density at the putative phosphate binding site compatible with a chloride ion [22]. The density observed for the Pi in the 1.8 Å structure presented here is clearly tetrahedral (proving that Pi and not Cl^−^ is bound) and facilitates accurate refinement of the four oxygen atom positions of the phosphate as well as determination of binding geometry (Figure 2(a)). The small error of only 0.1 Å in coordinal positions for this structure (as estimated by the refinement program REFMAC) allows for accurate determination of the hydrogen bonding distances (Figure 2(b)). This analysis reveals that the Pi ion is bound by four residues from the Rrp41 subunit. Specifically, the phosphate is coordinated by the side chains of R99, R139, and S138 as well as the main chain amino groups of G137, S138, and R139 (numbering according to the *S. solfataricus* sequence). A total of eight contacts with binding distances of 2.6–3.2 Å are observed creating a strong phosphate anion binding site (Figure 2(b)). The only previously determined structure of an archaeal exosome in complex with phosphate is the 2.65 Å resolution structure of the *M. thermautotrophicus* RNase PH ring [25]. Comparison of this structure with the *S. solfataricus* exosome structure presented here reveals a high structural similarity with an RMSD of 1.2 Å after the two structures are superimposed. The two phosphate binding sites are almost identical and reveal very similar interactions between the protein and the phosphate (Figure 2(c)). The only significant difference is the presence of a threonine in *M. thermautotrophicus* Rrp41 (T136) in place of the *S. solfataricus* Rrp41 serine (S138). However, in both cases the side chain hydroxyl interacts with the Pi ion. The position of the Pi-binding site is highly conserved between archaeal exosomes and RNase PH [32] and PNPase enzymes [20] indicating a conserved mechanism of phosphate-dependent RNA degradation among phosphorolytic exosome-like complexes.

### 3.2. Structure of the 9-Subunit Archaeal Exosome Bound to RNA and Pi

**Figure 3:**
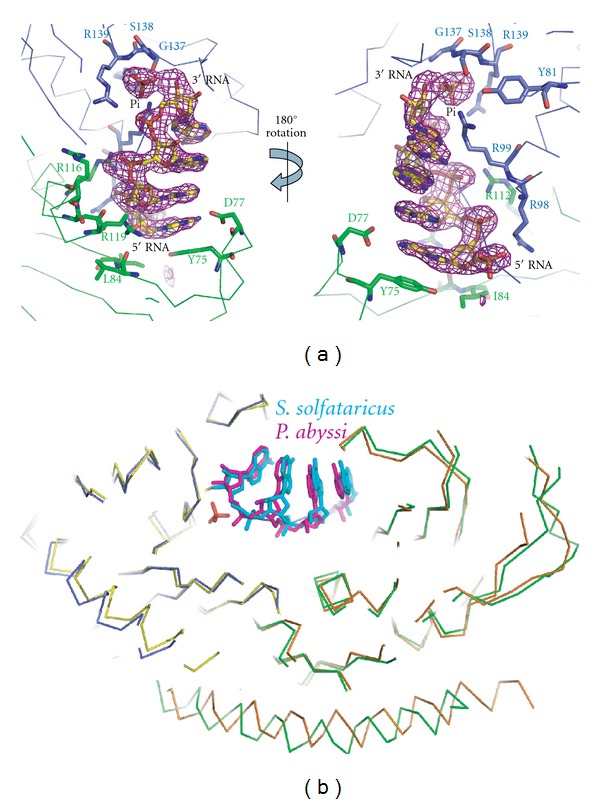
Detailed view of the *S. solfataricus* exosome active site in complex with RNA and inorganic phosphate. (a) RNA, Pi and contacting residues are shown as sticks (blue for Rrp41 residues and green for Rrp42 residues). An unbiased Fo-Fc electron density map at 3 sigma is displayed in magenta. Two orientations related by a 180 degree rotation around the vertical axis are shown. (b) Position of RNA substrates in the active sites of exosomes from *S. solfataricus* (RNA displayed in cyan, Rrp41 in blue and Rrp42 in green) and *P. abyssi* (RNA in magenta, Rrp41 in yellow and Rrp42 in orange) after superimposing the backbone C-alpha atoms. The position of the inorganic phosphate as observed in the *S. solfataricus *exosome is shown in red. The position of the RNA is very similar in the two different archaeal exosomes.

**Figure 4:**
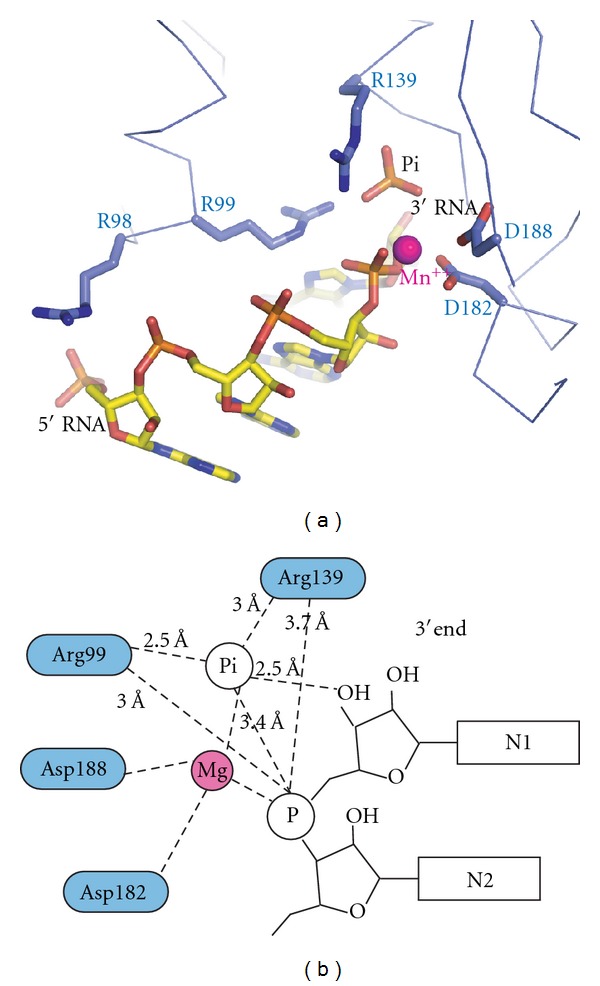
Model of the archaeal exosome bound to RNA, Pi, and Mn^++^. (a) The model shows the active site of the archaea exosome (*S. solfataricus *coordinates from this study) with a divalent cation (magenta ball) from *E. coli* PNPase (pdb code 3GME) after superimposing the coordinating aspartate residues. (b) Schematics of the model shown in (a) with interaction distances indicated as derived from the structure with RNA∗Pi bound presented here. A magnesium ion instead of a manganese ion is shown as the *S. solfataricus* exosome is known to be significantly more active with magnesium [26]. The divalent cation is positioned between the Pi and the 3′-end phosphate of the RNA but accurate coordination distances are not known.

The 2.5 Å difference map obtained from *S. solfataricus* exosome crystals soaked with RNA for 48 h followed by a quick Pi soak displayed clear density for the 4 most 3′-end nt of RNA as well as one Pi ion revealing a pre-catalytic state with both substrates bound (Figure 3(a)). The RNA binding mode is similar to previously structures of the *S. solfataricus *Rrp41/42 RNase PH ring (not shown) and *P. abyssi* Rrp41/42 in the absence of Pi (Figure 3(b)) confirming that mainly sequence-independent phosphate-backbone interactions hold the RNA substrate in place. Phosphorolytic degradation of RNA requires the nucleophilic attack of a negatively charged Pi on the negatively charged phosphate of the most 3′-end nt of the RNA substrate. In the structure presented here, the Pi ion is positioned only 3.4 Å from the 3′-end phosphate of the RNA substrate. The phosphorolytic mechanism of the archaeal exosome results in the build up of negative charge that needs to be neutralized. The close proximity of the phosphates is facilitated by two conserved arginine residues (R139 and R99 of *S. solfataricus* Rrp41) that neutralize the charge of the phosphates (Figure 4). Only smaller conformational changes in active residues of maximal 0.8 Å occur upon RNA binding in the RNA∗Pi-bound structure as compared to the Pi-bound structure. Interestingly, the 3′-OH of the 3′-end ribose of the RNA makes a close 2.5 Å contact with the Pi anion (Figure 3(a)) suggesting that the nucleophilic attack of the Pi may be RNA-substrate-assisted. From the electron density and the distance of 3.4 Å between the Pi ion and the phosphate of the 3′ end of the RNA substrate, it is clear that a nucleophilic attack has not yet taken place. As the structure presented here is of the Rrp41^D182A^ mutant, we conclude that D182 is required for initiation of the nucleophilic attack in the phosphorolytic mechanism of the archaeal exosome (further discussed in the following section).

### 3.3. Structural Model of the Archaeal Exosome with RNA, Pi, and Divalent Cations

The structural data presented here suggest that D182 of Rrp41 is required for the initial step of the phosphorolytic mechanism, namely, the nucleophilic attack by the Pi ion on the 3′-end nt of the RNA substrate. Based on the 3 Å resolution structures of the hexameric RNase PH core of the *S. solfataricus* exosome bound to RNA, it was suggested that D182 could serve the role as a general base by donating a proton required to form the hydroxyl-group of the 3′-end of the RNA substrate after nt cleavage [22]. This prediction was mainly made based on the geometry of the active site and the fact that mutation of D182 renders the enzyme completely inactive. However, in light of the new structural data, a role for D182 as a general base in the reaction mechanism seems unlikely. Rather, the function of D182 is, together with D188, to coordinate a divalent magnesium ion required for catalysis. Biochemical analysis of the *S. solfataricus* exosome demonstrates that magnesium is indeed required for both the degradation and the polyadenylation activities of the archaeal exosome [26]. Additionally, structural analysis of the *E. coli* PNPase in complex with manganese ions demonstrate the existence of a divalent cation binding site coordinated by residues equivalent to D182 and D188 of *S. solfataricus* Rrp41 [33]. A crystal structure is also available for the *S. solfataricus* exosome in complex with manganese but only for the Rrp41^D182A^ mutant in which the divalent cation binding site of the active site is disrupted [4].

By superimposing the structures of Rrp4/Rrp41^D182A^/Rrp42 bound to Pi and RNA presented here with wild-type archaeal exosome and *E. coli* PNPase bound to manganese ions, a structural model for the presumably fully functional phosphorolytic active site of archaeal-like exosome complexes can be obtained (Figure 4(a)). In this model, the divalent cation ion is located between the Pi and the 3′ phosphate of the RNA substrate and thus in a perfect position to facilitate the nucleophilic attack by stabilizing the pentacovalent intermediate state of the phosphorolytic reaction mechanism (Figure 4). This model agrees well with that of the proposed transition state of *E. coli* PNPase [33]. As no active site residue appears to be in a suitable position to act as the general base required for the protonation of the newly formed 3′-end upon phosphorolytic cleavage of the RNA, this role is likely served by water molecules present in the active site. Structural analysis of a pentacovalent intermediate state of the reaction mechanism would shed more light on this issue. Given the large degree of active site conservation between PNPases and the archaeal exosome, these complexes likely share a common reaction mechanism that relies on conserved arginines and a conserved Mg^++^ binding site to lower the energy of the transition state to facilitate phosphorolytic degradation of RNA. Given the loss of phosphorolytic activity of most eukaryotic exosomes, it appears that the archaeal exosome from a functional perspective is much more similar to bacterial PNPases than to eukaryotic exosomes.

## Figures and Tables

**Table 1:** Data collection and refinement statistics.

Rrp4/Rrp41^D182A^/Rrp42 space group P213	Pi	RNA ∗ Pi
Data collection		
Wavelength (Å)	0.98012	0.97995
Unit cell (Å), *a* = *b* = *c*	135.1	134.8
Resolution (Å)	40–1.8 (1.9–1.8)	40–2.5 (2.65–2.50)
*R*_sym_	0.082 (0.653)	0.125 (0.722)
*I*/*σ*(*I*)	11.4 (1.6)	14.5 (2.9)
Completeness	0.973 (0.901)	0.999 (1.00)
Redundancy	5.0 (2.7)	7.1 (7.1)

Refinement		
Resolution (Å)	40–1.8 (1.9–1.8)	40–2.5 (2.6–2.5)
No. of reflections	71709	28497
*R*_work_	0.183 (0.346)	0.191 (0.325)
*R*_free_	0.221 (0.375)	0.255 (0.376)
R.m.s deviations		
Bond lengths (Å)	0.017	0.009
Bond angles (°)	1.6	1.5
PDB code	4ba1	4ba2

Highest-resolution shell is shown in parenthesis.
